# Family Physicians’ Views of Who They Are Accountable To and Current Quality Metrics

**DOI:** 10.1001/jamanetworkopen.2026.9281

**Published:** 2026-04-16

**Authors:** Richard A. Young, Sandra K. Burge, Katherine S. Bergs

**Affiliations:** 1 Residency Program, JPS Hospital Family Medicine, Fort Worth, Texas; 2 Department of Family and Community Medicine, University of Texas Health San Antonio; 3 Behavioral Medicine in Primary Care, JPS Hospital Family Medicine, Fort Worth, Texas

## Abstract

**Importance:**

Primary care physicians are expected to demonstrate accountability for their work to health care administrators.

**Objectives:**

To determine family physicians’ views about common approaches to demonstrate accountability to health care administrators and for physicians who desire changes, to ascertain those proposed changes.

**Design, Setting, and Participants:**

In this qualitative study, semistructured focus group interviews were conducted from November 2021 to March 2024 in primary care offices of purposively sampled family physicians who concurrently saw patients in 5 different cities in Texas.

**Exposure:**

Interviewers queried participants about their views on existing accountability approaches by health care administrators and their views on improving accountability systems.

**Main Outcomes And Measures:**

Codebook thematic analysis identified themes characterizing participant views on demonstrating accountability to health care administrators, common quality metrics, and suggestions to improve accountability approaches.

**Results:**

Thirty-six participants were interviewed in 12 focus groups (15 [42%] were aged 31-39 years, 20 [56%] were female, and 20 [56%] were residency or medical school faculty). Significant themes included that participants were accountable to multiple stakeholders, and all stakeholders should be held equally accountable for their work. Existing quality metrics were perceived as useful tools to improve preventive and chronic disease goals for patient populations. Concerns about common quality metrics included that they sometimes contradicted patients’ wishes, were sometimes inappropriate, were sometimes corrupted by erroneous data, expected the physicians to control patient behaviors they could not, and penalized physicians who care for vulnerable populations. Participants reported moral distress caused by tensions between the demands of the metrics vs patient-centric care and their professional duties. Many participants had suggestions for improvement for assessing the quality and accountability of their work.

**Conclusions and Relevance:**

In this qualitative study of family physicians’ beliefs about accountability and common quality metrics, no participant was fully satisfied with the existing approaches for family physicians to demonstrate accountability for their work to health care administrators. Based on their responses, 2 proposals were created for a better approach to primary care accountability: minor adjustments that preserve some of the existing systems and major changes that represent a paradigm shift in the use of metrics to evaluate the work of primary care physicians and teams.

## Key Points

**Question:**

What are primary care physicians’ views on current approaches to demonstrate accountability for their work to health care administrators, and what changes would they like to see in the common quality metrics?

**Findings:**

In this qualitative study, 36 family physicians in focus groups said they were accountable to many stakeholders and that all stakeholders in the health care system should be held equally accountable. Participants reported moral distress created by conflicts between common quality metrics and patient-centric care and called for many changes to existing approaches.

**Meaning:**

These findings suggest that assessments of the quality of primary care work should be reimagined by all stakeholders.

## Teaser

This qualitative study evaluates family physicians’ views about common approaches to demonstrate accountability to health care administrators.

## Introduction

Primary care physicians report on the quality of their clinical work to health care administrators (payers and regulators) commonly based on a set of metrics guided by single-disease performance measures. The alphabet soup of these performance assessment approaches includes the Health Employer Data Information Set (HEDIS), Merit-Based Incentive Payment System (MIPS), alternative payment models including accountable care organizations (ACOs) and Medicare’s Comprehensive Primary Care Plus program (CPC+), and value-based payment plans (VBP) that are often tied to Medicare Advantage plans.

However, many metrics measure care processes–not physicians’ actions—and do not account for complex chronic illnesses, social determinants of health, or other factors that could negatively affect a patient’s health.^1,2,3^ These scores can be an inaccurate determination of the care provided by a physician and team.^4^ Challenges in reporting the quality of family physicians’ work through billing or electronic health record (EHR) data have been recognized previously.^5^ Others have criticized these monitoring programs for actually inflating health care costs^6^ and punishing physicians who care for disadvantaged patients.^7^

The Center for Medicare and Medicaid Services created the Meaningful Measures Initiative in 2017 in part because of physician complaints of the excessive administrative burden of collecting metrics data.^8^ These burdens may contribute to moral distress, which occurs when professionals fail to carry out what they believe to be ethically appropriate actions because of either internal or external constraints.^9^

Our qualitative study assessed family physicians’ views on demonstrating accountability for their work to health care administrators. We aimed to determine what frontline family physicians do and do not like about current approaches for demonstrating accountability to health care administrators, understand the role of quality metrics, and for physicians who desire changes, ascertain their proposed changes.

## Methods

This descriptive and inductive qualitative study of semistructured interview questions was performed using codebook thematic analysis to investigate family physicians’ views.^10^ Codebook thematic analysis uses a structured approach to coding without reliability tests, recognizes that some themes may have been predetermined before analysis, and accepts some researcher subjectivity. This study followed the Standards for Reporting Qualitative Research (SRQR) guideline. The study was approved by the University of Texas Health San Antonio institutional review board. Verbal consent was obtained from all participants.

Participants were purposively invited to represent a variety of practice settings. They were family physicians who identified as working in a primary care setting and who were currently seeing patients. They were in practice at least 1 year beyond their residency to ensure experience with quality metrics. Residency and medical school faculty were included, although to be eligible to participate, they had to see patients directly and have patient panels they were responsible for. Participants also included community physicians from private practice. Residency Research Network of Texas (RRNeT) members in 5 geographically dispersed cities enrolled local physicians based on personal relationships. Otherwise, no other preselection criteria were applied.

Focus group interviews took place from November 2021 to March 2024 (2.5 years were needed because of post–COVID-19 challenges and personnel changes in RRNeT). A study information sheet was given to each participant before data were collected and consent was obtained orally. Participants were given a brief demographic survey. They defined their race and ethnicity. Each interview followed a semistructured interview guide that was slightly adjusted after the first 2 sessions. HEDIS measures became deemphasized because younger participants had never heard of them. More recent metrics such as MIPS and value-based payment were included. Focus groups were conducted in person, over Zoom, or over telephone. The interview guide included questions about participants’ definition of accountability, what they liked and disliked about current metrics used to evaluate the quality of their work, and suggestions for a better accountability system. All sessions were audio-recorded, deidentified, and transcribed.

### Data Analysis

Transcripts were analyzed using codebook thematic analysis. Before analysis, the research team anticipated that themes would include comfort and concerns with current metrics. This understanding came from knowledge of the literature and personal experience.^4,11,12^ All members of the research team worked in family medicine departments for many years and had been present in meetings discussing these concepts.

Codes were hand-written on paper copies of the transcripts. R.Y. and S.B. created a coding structure independently and compared findings of major codes after 3 transcripts. Major codes were defined, and the remaining transcripts were coded and subcoded. The researchers discussed codes and themes several times during the course of the study, including K.B. to add another perspective. Consistent with codebook analysis, interrater reliability was not calculated. Themes were developed iteratively. Further details of our methods are in the eMethods in Supplement 1.

## Results

We interviewed 36 participants in 12 focus groups (15 [42%] were aged 31-39 years, 20 [56%] were female, and 20 [56%] were residency or medical school faculty). Demographics of the participants are shown in Table 1. When asked what accountability means, one primary theme was that physicians were accountable to many stakeholders. Further details of these results are in the eAppendix in Supplement 1.

**Table 1.** Participant Demographics

Characteristic	Participants, No. (%) (N = 36)
Sex	
Female	20 (56)
Male	16 (44)
Race and ethnicity	
African American or Black	1 (3)
Asian	8 (22)
Hispanic or Latinx	8 (22)
White	18 (50)
Work site	
Residency or medical school	20 (56)
Private practice	16 (44)
Degree	
MD	28 (78)
DO	8 (22)
Age, y	
31-39	15 (42)
40-49	13 (36)
≥50	8 (22)
Years in practice	
2-5	10 (28)
6-10	12 (33)
11-20	9 (25)
≥21	5 (14)
Other characteristics of participants, mean (range)	
Panel size	1750 (150-4000)
Patients seen per wk	98 (10-650)

Abbreviations: DO, doctor of osteopathic medicine; MD, doctor of medicine.

### Stakeholder Accountability

The first primary theme was that there should be equal accountability for all stakeholders (eg, hospitals, insurance companies, specialists, and patients). Participants said accountability focused only on physicians was not appropriate. Accountability should work both ways, with patients and with health care administrators. Data should be linked across systems and physicians should not be expected to fill in data gaps. “I remember that ‘ah-ha’ moment when I realized there’s Venn diagram between the payer, the physician, and the patient, and there’s a very, very small piece where all three are like on the same page. But, it’s very disconcerting that we’ve got this sort of third piece in the room which is the payer, which is not the patient.”

Some participants said patients should be incentivized to achieve metric outcomes. “I feel like the insurers should do a better job of incentivizing patients to have a relationship with a primary care physician and disincentivizing the other, going to the dermatologist.” Others thought the insurance companies should not contact physicians when metrics were not achieved. “The insurance company always sends us the statements like, ‘Your patient has not been consistent with their antihypertensive, haven’t filled their statin in three months.’ Like, why are you mailing this to me? Why don’t you call them and tell them, ‘You need to go see your doctor?’”

### Quality Metrics

#### Quality of Care

The second primary theme was that quality metrics results do not always equate to higher quality of care. Many participants believed that multiple factors influenced whether a specific patient could reasonably meet a metric, and standard metrics were not a reflection of the quality of work by the physician or team. “I just don’t think there’s a single, or even a group of measures that’s gonna accurately reflect how good a doctor is based on some boxes that you can check…from afar on the computer.”

Examples of complicating factors included patient preferences, how long the patient had been seeing that physician, comorbidities, patient age, and many social determinant challenges to patients including built environment, cultural factors such as diets, the lack of health insurance, and the high price of medications, especially insulin. “I don’t need certain measures to tell me, like, how I’m doing…. [T]hey don’t factor in the other complexities of patient care, people who can’t afford insulin, people who come to you once and never come back, people who just outright refuse to do something despite you…spending 15 minutes counseling them and they still don’t do it.”

#### Wasteful Care

A secondary theme under quality metrics was that such metrics can incentivize wasteful care. Participants expressed many concerns about quality metrics causing wasteful care. Metrics sometimes contradicted patients’ wishes or were inappropriate for some patients. “We measure these metrics on patients who are on dialysis…so you don’t need to continue to check and prove that they have chronic kidney disease. But yet...the metric says I have to do it. So, I’m going to order this test that I don’t really need. It’s not going to change my clinical decision making [nor] the outcome for the patient.”

#### Moral Distress

The third primary theme was that existing accountability approaches can cause moral distress to physicians. Participants said they are sometimes distressed by metrics that conflict with ethical patient-centered care. Examples of moral distress beyond the ones listed subsequently are located in Table 2.

**Table 2.** Reports of Moral Distress Caused by Current Accountability Approaches

Secondary themes	Brief supporting quotes
Metrics generate negative feedback to physicians that discount their efforts	“[I’m] feeling like [I’m] constantly getting negative feedback…because we have such a challenging patient population,…‘I’m trying. I’m doing everything I can.’ Unless these people move in with [me].”
Metrics create undue guilt for physicians	“Part of the burnout is like a little bit of guilt that comes along with when your patients aren’t at goal, or when you don’t have them up to date with their mammogram and their colonoscopy and everything. Then you’re like, ‘I’m doing the best that I can.’”
Metrics decrease the joy of meeting a new patient	“70% of my patients are new. So you come in and you say, ’What? Where’s your colonoscopy, mammogram? Did you do your pap smear?’…And they go, ‘Uh I don’t have a job.’… Because they don’t have the copay,…now I know their [hemoglobin] A_1C_ is going to be over 8.”
Metrics do not evolve, nor acknowledge professional differences of opinion on the standard of care	“Most of the metrics…don’t change as quickly as guidelines and medical knowledge change.… ‘[Y]our [hemoglobin] A_1C_ rates are not great.’… [But] actually their goal is 7.5.… And your blood pressure, I mean everyone is still arguing about what an actual controlled blood pressure is. Stop holding me to a standard when we haven’t even been able to define a standard yet.”
There is no accountability of insurance companies, pharmaceutical companies, and hospitals to patients; and physicians feel helpless to do anything about it	“And I think there’s no accountability for hospital systems, and what they do for how they handle uninsured patients. How they bill uninsured patients at a much higher rate that has no comparison to what they would ever get from insured patients.… [W]e cannot put forth any accountability towards hospital system because they’re so powerful, and the insurance companies and pharmaceutical companies, and hospitals are so powerful, there’s no way to hold them accountable.”
Patient experience scores are likely to be biased toward outliers	“The people that have filled out surveys are the people that we know really love us or the people that are really angry and so, I feel like it’s a poor selection size, right?… [T]hat doesn’t make me a better physician. It just makes me frustrated or makes me jaded.”
Patient experience scores incentivize inappropriate prescribing	“[It] pushes you to give the patient something that you probably wouldn’t do just because you’re trying to keep satisfaction scores really high.… I feel like you see a lot of inappropriate antibiotic use because of that,…more controlled substances than you probably want to do.”
Patient experience scores incentivize inaccurate diagnoses	“And so there were providers…that inappropriately put the diagnosis of diabetes, even though they didn’t meet any criteria, just to appease patients and get GLP-1s like semaglutide or Mounjaro covered.”
Metrics goals can be about profit for a health care system rather than improving quality	“I guess this is really not as much about how good of a doctor you are, but more they did just check, how many in-network referrals you do for cardiology, GI. You had to be above a certain percentage, you know, and not offering outside [referrals].”
Value-based payment incentives increase note bloat and take the focus away from other aspects of patient care	“[T]hey’re really pushing for HCC coding, was like kind of a burden as well as being a little bit inappropriate. It caused a lot of providers to put probably not true diagnoses that they would meet criteria for, but…that made the patient look more complex, that gave, you know, better reimbursement, better risk adjusted scores.… Then you just have these huge bloated notes in which 15 problems were on there, but you didn’t really address anything. You just put continue to monitor.”
Frustrations about the existing state of primary care lead to participants advising their children not to go into medicine	“It’s a system, and…it really saddens me. And I love my job and I love seeing patients and I would never do anything else. But I mean, I have so many friends that way and they always say, ’I don’t know if I want my child to go into medicine, even if they’re interested.’ I mean, it is such a burden.”

Abbreviations: GLP-1, glucagon-like peptide; GI, gastroenterology; HCC, hierarchical condition categories.

##### Conflicts With Patients, Other Stakeholders, and the Physicians’ Sense of Professional Duty

A secondary theme under moral distress was that pressure to achieve metrics creates conflicts with patients, other stakeholders, and the physicians’ sense of professional duty. Participants wanted to care for patients in a manner that respected patients’ wishes and unique preferences but then felt penalized by incentives or disincentives embedded in the metrics. “We get lists of patients that haven’t filled their medications, and they’re going to hit a gap,…So, we call patients and remind them that they need to go pick up their medications. If they tell us they have enough on hand, that they are not out, we just tell them, ‘Look, please just go pick it up,’…so that you’re not missing this measure.… That’s not fair that I don’t get paid because they’re not going to take their medication.”

Accountability metrics can also put physicians in conflict with their clinic administration, leading to pressure to practice inappropriate medicine, especially related to patient experience measures. “The patient was expecting a Rocephin injection and I didn’t give it to them [for a viral illness].… [T]he patient called the office manager, and complained, right? So the office manager comes to me and lets me know this is what the patient paid for, and can I give the patient the Rocephin shot?”

##### Time Spent on Patients’ Concerns

Another secondary theme under moral distress was that existing accountability approaches force physicians to spend less time on patients’ concerns. Current accountability approaches can change the nature of an office visit, prioritizing metric goals over patients’ agendas. “I think we spent almost 80% of the patient visit just trying to deal with the metrics. And only 20% really goes into true patient care, listening to what’s going on with the patient, evaluating their real problems. The primary goal is to meet the metrics now, and that’s not good medicine.”

##### Penalizing Physicians Who Care for Vulnerable Populations

A final secondary theme under moral distress was that existing accountability approaches penalize physicians who care for vulnerable populations. Current accountability approaches penalize physicians who care for vulnerable populations, such as low-income patients whose care is made more complex by a variety of social determinant challenges. “[M]ost of our patients are self-pay.… I want to have better quality, increased quality medicine, I can’t. They don’t have any money to pay it. So, on the paper it looks like I’m not doing a good job,…There’s no like, checkbox, ‘patient can’t afford colonoscopy,’…or ‘patient can’t afford ophthalmology.’”

#### Better Accountability Approaches

##### More Resources for Existing Accountability Approaches

Under the primary theme of better accountability approaches, some participants wanted more resources for existing accountability approaches. When asked “What would a better accountability system look like?” some respondents first suggested a wish list of additional resources to help them meet the existing accountability approaches, such as social workers, data experts, and more help from health care administrators. “I would ask for a social worker in my clinic and try to emulate other clinics like [X] Clinic. They have this pharmacy there and that will help me a lot, at least with the insulin.… And, well the social worker…and training for the MAs.”

##### Overlap Between Value of Work and Common Metrics

Another secondary theme under better accountability approaches was that some participants acknowledged the overlap between the value of the work they do and common metrics. Some felt that the existing metrics represented a reasonable reflection of their work and just needed some adjustments. When asked what work they did that was valuable but not reflected in the current metrics, some respondents did not suggest many changes. “I think they’re good. I think they are there for a reason. I don’t disagree that we should be screening these patients.”

A common suggestion for change was that physicians should be recognized for effort and not be penalized for outcomes they could not control. “If you’re counseling a patient to exercise 30 minutes a day, five days a week, and they’re not doing that, we’ve documented it, and that documentation should suffice for a patient’s poor outcome.” Some suggested creating more checkboxes. “Instead of having a checkbox ‘have done’ ‘not done,’ have another checkbox ‘not done because of cost’ or ‘not done because of personal preference.’”

Participants said health care administrators should reward physicians for improvement in metrics, even if 100% is not achieved, keep targets constant, and avoid target creep. “Quit making the metrics so hard to achieve. Take it at nine and then leave it there. Don’t make it harder and harder and harder every year.”

##### Flaws in Common Accountability Approaches

A final secondary theme under better accountability approaches was that some participants felt that common accountability approaches were severely flawed and wanted major changes. Some participants said metrics are too flawed to be used. “I don’t think we need to have quality metrics. I do think that it can be good for the patient, but I think that it should be the patient’s decision whether they want to do these things.” Participants had concerns about bad data being used in quality assessments. Some believed no valid adjustments exist for physicians who care for vulnerable populations, and many were concerned that metrics goals can ignore patients’ wishes.

Some participants liked the metrics to serve as internal reminders but said they should not be used for ratings or payment. They would mostly use internal metrics to identify physicians who are extreme outliers. “That’s what’s going to define who the outliers are and where they should be targeted to, right?… You’re going to get tails on both ends….[E]verybody wants an ‘A’ doctor. It’s like no, we want to make sure that aren’t some ‘F’ and maybe some ‘D’ doctors out there.” These participants also suggested that health care administrators should see that physicians do their own peer review and reward them for that monitoring. “And as a profession, if we don’t police each other and police ourselves, somebody else is going to feel the need to step into police us. You just have to leave the As versus the Bs versus the B minuses to the peer review level, you know? Focus on the Ds and the Fs [with quantitative metrics].”

Some participants suggested eliminating patient experience as a metric, saying patients will vote with their feet. “Someone’s not going to come back to you if they thought you’re doing a bad job.”

Participants offered solutions to imagine a better system for family physicians to demonstrate their accountability to their multiple stakeholders. We summarize their suggestions for a better accountability system that could include (1) minor adjustments to existing processes that retain some of the current features of existing accountability approaches, or (2) major changes that represent a paradigm shift in the understanding of the place of current metrics in primary care, based on a belief that what is measured is not necessarily meaningful, and what is meaningful cannot be easily measured^13^ (Box).

Box. **Solutions for a Better Accountability Approach With Both Minor Adjustments to Existing Processes and Major Changes**

**Minor adjustments to existing processes suggested by participants.**

**Common metrics**

- Keep the current chronic disease and preventive metrics, but create additional reporting for exceptions (patient declines service, cannot afford service, comorbidities, and so on).
- Measure improvement over time, not just meeting or not goal values.
- Align metrics to patient goals.
- Periodically measure continuity patients on the common preventive and chronic disease services, and be credited for the work. Physicians should control when they:
  - Offer a test or treatment.
  - Counsel a patient to make a behavior change.
  - Refer a patient to the next service practitioner, such as an eye examination.
- Reward practices that have EMR-based reminder functions.
- Add new metrics:
  - Patient retention.
  - Quality of physician-patient relationship.
  - Measurement of patient efforts.
  - Interpreting specialists’ explanations into more patient-appropriate language.
  - Coordinating care such as doing work generated for primary care by specialists such as FMLA paperwork or ordering services recommended by a specialist.
  - Screening for social determinant factors such as food insecurity.
  - No-show rates, adjusted for characteristics of patient panel.
  - Anxiety or depression management.
  - Avoiding referrals to specialists.
  - Acute care measures.
  - Work performed to help patients navigate the complex health care system.
- Set reasonable targets for practice outcomes, including RAF scores, that do not constantly creep upward; never 100%.
- For RAF scores, eliminate the triple score for hospital readmissions.
- Modify metrics goals in light of social determinant influences.
- Reward physicians for cost-effective decision-making (ie, not ordering low-value services).

**Patients**

- Create incentives for patients to achieve recommended metrics.
- Create better patient experience surveys: if not satisfied, why?

**Major changes: eliminate most data collection for and reporting to health care administrators.**

- Reward practices for periodically monitoring their regular patients for the common preventive and chronic disease services, but do not further reward and penalize the outcomes.
- Use basic EMR-enabled care management measurements to identify extreme outliers. The practice shows that it periodically assesses physician performance in this way and then dives deeper to understand why an individual physician’s performance looks so different from the rest of the group or common national norms. The individual physician or team numbers are not reported to health care administrators.
- Recognize that metric outcomes are too dependent on patient factors, system barriers, and social determinants of health realities to ever be used to evaluate the quality of family physician or teamwork.

**Patient experience**

- Do not measure and report this, nor reward and punish physicians for the results. Patients will vote with their feet.
- Individual physicians or practices may want to measure patient experience as a tool to identify unknown imperfections in their clinic and for individual physicians to self-reflect on their patient care style.

**Other physician work**

- Eliminate CMS documentation rules and regulations for primary care.
- Recognize and reward periodic subjective peer-to-peer assessments using medical record reviews within group practices. Only physicians can comment on the work of other physicians.

**Health care administrators**

- No physician accountability metrics, incentives, disincentives without similar actions for patients, payers, and regulators.

Abbreviations: CMS, Center for Medicare and Medicaid Services; EMR, electronic medical record; FMLA, Family Medical Leave Act; RAF, risk-adjustment factor.

Other examples of themes, subthemes, and supporting quotes are included in the eTable in Supplement 1. We found no stark differences between the responses of the academic and private practice participants. The participants in residency-based practices perhaps discussed more the challenges of caring for vulnerable populations. The participants in private practices discussed more their challenges of meeting the expectations of value-based care or Medicare Advantage plans.

## Discussion

This qualitative study found that family physicians were comfortable with being held accountable for the quality of their work to health care administrators, but they also said they were accountable to many other stakeholders, and sometimes there were conflicts between these goals. All focus groups mentioned common quality metrics as being an important part of how they currently are asked to demonstrate the quality of their care to health care administrators. Many were unhappy with the current system of accountability and its associated metrics. To some, the metrics included common tests and treatments that they thought were important in their direct patient care, and they had few suggestions for change beyond the common metrics. Others thought there were too many flaws in the metrics to be meaningful and called for their elimination as tools for health care administrators to judge the quality of work of family physicians.

Participants in residency clinics discussed the challenges of caring for vulnerable populations. This is consistent with previous research that estimated that sociodemographic factors accounted for as much as 25% to 50% of the observed variance in measures such as hemoglobin A_1C_ or blood pressure control.^11^ The lack of a proven way to adjust for the differences in the capacities of different patient work systems was a consistent frustration, which was similar to previous research demonstrating the challenges of meeting standard metrics in Medicaid-funded and uninsured patients.^14,15^

Our findings are consistent with previous research in several domains: frustration from the amount of clerical work in EHRs^16^; factors such as paperwork, feeling undervalued, frustrations with referral networks, and difficult patient situations^17^; and observing that one metric outcome may be only loosely correlated with performance in other metrics.^18^ Participants also said that the complexity of primary care is not acknowledged in the metrics^19,20,21,22,23^; that the current quality metrics may not reflect the quality of care a primary care physician provides^2,24,25^; there may be value in some of the metrics,^26^ but the metrics should be used as a tool of practice self-reflection, not to be judged by outsiders nor tied to payment.

Our study adds to the literature on moral distress or injury. Previous research identified factors that impede family physicians’ ability to provide needed care to their patients such as a limited ability to set boundaries at work, payment policies, scope of practice, the availability of social welfare programs, being challenged hospitals’ culture of consumerism and families’ expectations for treatment intensity, feeling disempowered by medical hierarchies, and generally being demoralized by the overall health care system.^27,28,29,30^ Our study adds to this literature, particularly in the tensions between the physicians’ judgment on the best patient care or most patient-centric patient care (ie, respecting their wishes to not receive certain services) vs single disease measurements. This tension was particularly heightened when their professional ethics were in conflict with patient experience scores.

Our participants were not the first to question the use of metrics and, for some, to question whether they have any real value. A recent editorial concluded that, “These well-intentioned efforts to improve quality have failed. Instead, we have seen the emergence of a quality industrial complex—a bureaucratic amalgamation of health care providers, regulators, and the private companies that service the health care sector.”^31^

Given the reported failure of the metric-driven patient-centered medical home effort,^32,33^ using single-disease metrics and patient experience scores for recognition and payment in primary care may be a suboptimal approach. The CMS Meaningful Measures initiative aims to “reduce the administrative burdens on healthcare providers while focusing on high-priority quality measures that truly improve patient outcomes.”^8^ This initiative is not focused on primary care and there has been little published in the peer-reviewed medical literature on its results.^34^ Our participants implied that they have not seen meaningful changes from this program, but in this study, they provide their own suggestions for better ways forward.

### Limitations

Limitations of our study included the purposive sampling of our focus groups. It is possible that physicians outside of RRNeT’s colleagues would have contributed other perspectives. However, we recruited family physicians from a variety of financial structures: residency faculty, medical school faculty, and the private practices of both independent and employer ownership models. Another limitation is that we only obtained the perspectives of family physicians known to RRNeT members, not other primary care physicians or advance practice professionals, or other stakeholders such as patients and health care system leaders. Other limitations include having only 1 physician participating in the analysis who had published in this space before, and the 3-year time frame of our interviews. Our original assumptions may have influenced findings; we started the project assuming HEDIS, MIPS, MACRA, CPC+, and ACO environments would be the topics for our interviews. In later interviews, more of the realities of Medicare Advantage contracts were discussed, such as hierarchical condition categories and risk adjustment scores. The overall themes were still very similar across the time frame of our study.

## Conclusions

In this qualitative study of family physicians’ beliefs about accountability and common quality metrics, no participant was fully satisfied with the existing approaches for primary care to demonstrate accountability for their work to health care administrators. Based on their responses, the researchers created 2 proposals for a better approach to primary care accountability: minor adjustments that preserve some of the existing systems and major changes that represent a paradigm shift in the use of metrics to evaluate the work of primary care physicians and teams. We challenge health care administrators to reimagine primary care accountability and performance metrics, to better balance accountability expectations for all stakeholders in the health care system, and to codesign with primary care a more meaningful system that recognizes the complexity of patient-centric primary care.

## Notes

Supplement 1. eMethods. eAppendix. Supplemental Results eTable. Additional Identified Themes, Subthemes, and Supporting Quotes

Supplement 2. Nonauthor Collaborators

Supplement 3. Data Sharing Statement
