# Supplementary material for: Family Physicians’ Views of Who They Are Accountable To and Current Quality Metrics
Source: JAMA Netw Open. 2026 Apr 16;9(4):e269281. doi: 10.1001/jamanetworkopen.2026.9281 (PMC13087814; doi:10.1001/jamanetworkopen.2026.9281)
Supplement: Supplement 1. — eMethods. eAppendix. Supplemental Results eTable. Additional Identified Themes, Subthemes, and Supporting Quotes [file jamanetwopen-e269281-s001.pdf]

Supplemental Online Content

Young R, Burge SK, Bergs KS. Family physicians' views of who they are accountable to and current quality metrics. *JAMA Netw Open*. 2026;9(4):e269281. doi:10.1001/jamanetworkopen.2026.9281

eMethods.

eAppendix. eResults

eTable. Additional Identified Themes, Subthemes, and Supporting Quotes

This supplemental material has been provided by the authors to give readers additional information about their work.

© 2026 Young R et al. *JAMA Network Open*.

eMethods.

RY is an academic family physician who had published work pointing out some challenges of quality metrics reporting for primary care.[10] He was aware of his pre-existing beliefs and sought co-researchers who may have fewer preconceived ideas about the issues studied. SB is a recently retired behavioral health faculty who had worked in academic family medicine for over 30 years. She was aware of some of the study issues but was never personally affected by them for outcomes such as job performance evaluations and income. KB is an academic psychologist at a family medicine residency whose job and income are not connected to most of the metrics of the physicians in her department.

Interview Guide

1. In today's environment we use the term "accountability" quite frequently.
    - What does accountability in medical practice mean to you?
    - What does accountability look like to you? Examples?
    - How are accountability and quality related or different?

Second, the questions will begin to focus more on experiences with measures:

2. How is accountability currently measured/gauged for you?
    - If you work in more than one practice, i.e. different environments- consider the questions from both perspectives.
3. How do these common measures such as HEDIS, MIPS, and MACRA impact your practice?
    - What about these measures do you agree with?
    - Do these measures inhibit your practice? Do you feel disincentivized?
    - Do measures burden you?
        1. Is it a time burden, effort burden, or?
4. Do these measures help make you a better or worse physician?
    - Examples (of both)?
    - What would you like to see measured or assessed because it would show that you are a really good doctor?
    - What would you like to see avoided?

Finally, questions will turn to asking physicians to project into the future and identify what they would envision accountability could look like in their practices:

5. How would you create a better accountability or quality system between physicians and payers/regulators?
6. In this new system, how would data be collected?
    - What is realistic in your practice setting?

© 2026 Young R et al. *JAMA Network Open*.

    - How often should data be collected?
7. Which of your measures or assessments (new) should be tied to payment?
8. Which current measures would you keep?
    - Is there anything else that you'd like to add? Any aspects of accountability or practice measures that I didn't mention?
9. How should Press Ganey type measures of patient satisfaction be part of accountability?
    - What do you think about online rating of physicians?

eAppendix. eResults

PRIMARY THEME: PRIMARY CARE PHYSICIANS ARE ACCOUNTABLE TO MANY STAKEHOLDERS

When asked what "accountability" means, physicians mentioned many different stakeholders: insurance companies, Medicare, patients, employers, colleagues, their practice and health system administrators, the state Medical Board, family medicine residents, medical students, best evidence, and themselves. The first reaction in most of the groups was payers and regulators, but some respondents made it a point to say that their first obligation was to the patient, *"My first accountability is to the patient, so I don't get quite as excited about external things as far as, you know, reimbursement or some of those things."* Other participants questioned if the primary care physician should be held accountable if a patient was seeing a specialist for a chronic disease, or if health system or insurance company barriers made it difficult for a patient to receive recommended services.

Secondary Theme: What are family physicians accountable for? Some existing metrics are reasonable.

All participants recognized that existing quality metrics were an attempt by payers and regulators to hold physicians accountable for quality of care. Many participants thought common preventive and chronic disease metrics were reasonable to improve the health of their patient populations. These service metrics included mammograms, Pap smears, cholesterol testing, hemoglobin A1Cs, blood pressures, and diabetic preventive services such as eye exams. Participants stated that the metrics and reminders built into their EMRs helped them be better physicians. *"[T]he reminders and all*

© 2026 Young R et al. *JAMA Network Open*.

that, it is helpful, and you don't want to miss something. If you're too focused on trying to get something else better, you don't want to miss their preventive care, or like, overlook something."

PRIMARY THEME: QUALITY METRICS RESULTS DO NOT ALWAYS EQUATE TO HIGHER QUALITY CARE

Participants said that it was common for data used to derive quality scores to be incomplete, and therefore the metrics did not accurately reflect the physicians' work. A lot of time and effort was spent trying to improve faulty data. *"If they got a colonoscopy somewhere else, then the insurance company should know that, and they should notify us. There should be like a universal bank that keeps track of all this, because half my time is spent like trying to get records from another doctor that they had their colonoscopy with and their pap smear."*

PRIMARY THEME: EXISTING ACCOUNTABILITY APPROACHES CAN CAUSE MORAL DISTRESS TO PHYSICIANS

Tension between physicians and patients can arise when accountability metrics pressure the physician to manage a patient in a restricted way. *"[W]hen you documented they have high blood pressure and at some point they've been on an ACE inhibitor or an ARB, if you for some reason take them off of that or they see the cardiologist and they stop that, put them on a beta blocker and that's all they're on, beta blocker doesn't count for the measure. So they're failing the … blood pressure medication [metric] because only ACEs and ARBs count for that measure."*

eTable. Additional Identified Themes, Subthemes, and Supporting Quotes

Themes	Sub-themes	Additional Quotes (Representative quotes are listed under results)
**Accountability – What are physicians accountable for?**		

© 2026 Young R et al. *JAMA Network Open*.

Common metrics are reasonable and help physicians deliver high-quality care for common chronic diseases and preventive services.	Metrics may reveal poor patterns of care	*"I get the value-based care mish-mash you know. You don't want people going year after year to their nurse practitioner or physician without things getting better. They're just getting paid by the visit. Preventive care gets overlooked. The treatment that's being provided isn't evidence based."*
	Metrics on EMRs create visual reminders that are helpful	*"I like the visual reminder that somebody is due for preventive measure, because in our very complicated patients, it's super easy to get lost in what's today's acute issue, right? And, I remember the moment as a young physician I realized that I had been seeing a patient monthly for her chronic issues involving pain medication, and every time she came in there was something she wanted to talk about. And so, I realized 18 months in we hadn't addressed her mammogram, and we hadn't addressed her colonoscopy. Because there was always something else. And so, it's nice to have a little bit of a reminder to go, 'Okay, I can chunk out this piece and this one."*
	Metrics make doctors become more involved with administration	*"[The measures] make me sign up for more committees, so I can try to make them go away or be mediated or be more understanding."*
Meeting metrics improve practice revenue.	Metrics may enhance revenue streams of one-off programs, even if the work asked by the metric may be thought of as meaningless.	*"No margin. No mission. Like, I understand the place has to get paid or I could never do anything. … Which of these is actually getting [the institution] millions of dollars and which of these is something someone made this up for a grant ten years ago and people forgot to get rid of it? And then I try to pass on that knowledge to people like, 'hey, I understand that check box is actually worth $1,000,000 with multiple examples this year.' A checkbox is worth a $1,000,000, and then something like this, I counsel them about their BMI, you know, smart phrase thing that you're supposed to click that is actually meaningless, right? …*

© 2026 Young R et al. *JAMA Network Open*.

		[T]he one that actually has huge financial impact gets lost in the noise.”
**Accountability – Metrics do not equal good care**		
Many participants expressed concerns about the metrics including they question their validity; create unrealistic expectations for patient choices physicians cannot control; decrease physician autonomy; decrease patient-centric care; create conflict between physicians and patients, and conflict with others on the team; demonstrate mistrust between payers/regulators and physicians; increase physician work, both reasonable and unreasonable; increase practice costs; and penalize physicians from caring for vulnerable patients.	Not clear why metrics chosen	*“My understanding or frustration with all those measures, HEDIS included, is that I’m always trying to figure out who made the decision as to what goes on that list and why? And, nobody can ever answer the question. So, it's always hard to tell, like, what state? Was it this insurance company? Was it that insurance company? Was it federal? Was it [the institution] just decided? Was it someone got a grant and they decided that we're going to put it on all systems?”*
	Metrics do not measure many important aspects of care	*“So other things that I see that … good docs should do … is specialty care coordination, referrals, home health. I mean no one looks at that, and think of all the time we do trying to make that happen. And of course, it's frustrating in our system. Just because you order it doesn't mean they're actually going to get their appointment and actually can be able to go. The evidence-based care is a hard one that would be another thing because oftentimes it's individualized as far as exactly what medication you have your patient on, exactly how you're treating them.”*
	Metrics are artificial without doctor-patient continuity	*“I think the metrics are artificial, and they are disheartening right now, because we can't maintain the continuity to even say who should own a bad number.”*
	Metrics penalize physicians for	*“And this is a good example of not being right with patient readmission. Last year it*

	actions taken by other doctors and facilities	*was kind of one time. This year it was counted three times. Well, I … have [patients] that come out of the hospital to skilled nursing. They don't get taken care of in skilled nursing and they get readmitted and I'm not part of that at all. And I get dinged.*
	Metrics create conflict with patient priorities	*"Patient experience. Which, ironically, is often at odds with check box quality measures in medicine. Right? My doctor did not look at me. They're looking at the computer. They didn't ask me about what I was interested in. They had this agenda. I came in for foot pain and they considered doing microalbumin, or pap smear."*
	Metrics decrease physician autonomy in those who work for large healthcare organizations	*"And that administrative attempt at helping people be efficient removes the autonomy, you know, from the doctor, right? That, well, maybe some women … feel comfortable when you walk in that they're already in a gown. But sometimes I've never met that patient and I'm a man walking in."*
	Metrics represent decreased trust in primary care physicians	*"(Dealing with outside report) - Or they could actually trust the physician to say, 'I saw the report. There it is, and now I can take it off of here.'"*
	Metrics cause physicians to work outside of office hours for patients who are not part of their practice	*"It's all the hours outside of work that we're running lists and go over the computer. Oh well, these people haven't been seen or, oh well, they don't have me as their primary anymore. They're seeing somebody else, but I can't get them off my panel. It's list after list, and there's such a pressure to try to get those done and get so many of those measures met. It's very stressful.*
	Metrics create unreasonable financial pressures on practices	*That is one reason why I feel hopeless is that, first of all, it we're looking at the problem I talked about earlier, incremental change. Well, sometimes when we try to measure incremental change, even that's a disaster. Like this year on women's health we had to show a 10% improvement in our rate of documentation for hemorrhage and our baseline was*

© 2026 Young R et al. *JAMA Network Open*.

		99 percent. And we literally got monthly remember emails that we had to get to 99.1 or we were going to lose out on millions of dollars of state funding.
	Metrics do not measure other important aspects of care	*"What would you like to see measured to show that you really are a good doctor? How much I care? (laughter) Well, what actually depends only on me. There are certain things that don't depend on me. Whether I could choose the right antibiotic for this or not the give antibiotic here. Or the right test here or not the test here. Or something like that, instead of something that I depend on the patient to go and complete."*
	Metrics are often not about the doctor work	*"I don't know that we've really tried hard enough to find things that are doctor dependent as opposed to patient dependent."*
	Metrics create non-sensical work	*(Female participant) "If that makes you feel better, I'm getting a PSA done on Friday because that's what the VA does for all of their patients. I'm curious to see the result."*<br>*(Another participant) "You're getting a PSA done?"*<br>*(Female participant) "Apparently, I'm getting one done because I asked her what she was putting in for me, and she said 'I'm putting in a PSA.' And, I said, 'a PSA, really?' 'Yes, it's on the list.'"*
	Many checkbox actions are often meaningless	*"[T]hink of all the charts that have documented 'more than 50% of this encounter spent counseling the patients,' like, seriously? Really? I mean people put that in there for, you know, very complete physical exams. Is that really, everybody actually checked all that stuff? So, it just becomes meaningless when you figure out how to game the system and that notes end up being templates, so that people get credit for things."*
	Metrics create conflict between	*"I think for a little while probably about two years ago we had whoever was rooming the patient automatically put in orders for*

© 2026 Young R et al. *JAMA Network Open*.

	primary care team members	*everything that was on the care gap list which was a pain, you know, having to go through and taking things off often. Or, … we're still signing a sheet that has everything listed on it, but frankly I just ignore it and sign it."*
	Metrics increase the work and administrative cost of primary care	*"I learned recently that we actually, if a patient has done say a mammogram, an outside mammogram and we have it, but it's not coming off the care gap, there's a super easy way to just screenshot, send it to an email, and there are people downstairs who are paid to update that for us so that it goes away off care gap and it doesn't keep being noise, right?"*
	Metrics shift work from support staff to physicians	*"It was someone else's job to pull the manila envelopes out of the thing and mine it and go back and ask, 'what you actually mean here?' and then submit it. And it took them all this time and then the EMR was able to either eliminate or redirect that person's workflow. And they said, well the physician can just do that with a couple clicks on front. The physician can order that pap smear with those 17 clicks about what the clinical history is instead of somebody else having to mine that information manually when they submit your labs. Like it always used to happen, it but it just never was the responsibility of the doctor."*
	Metrics create work creep – adding more work to do per visit	*"But going back to the signal versus the noise, it's like scope creep of what has, you know, the necessary has turned into. Well, if we just add this one more thing in the health maintenance [for a Medicare wellness visit]. … Well, that was just so the claim could make more money, right? Because we're trying to remind doctors to do something that makes money. … We're already doing this with our patients. It has no impact, right? So, the scope creep of what's required of us within these metrics is what frustrates me on a day to day basis."*

© 2026 Young R et al. *JAMA Network Open*.

	More efficient metric reporting may require more IT resources	*"Because I have worked in a system where we were allowed to just go ahead and say I did it and then the computer would auto populate things, you know on the time it was supposed to. Like, you say 'pap done' but if we put in the right diagnosis code, so that the computer then was able to say, 'Oh, no cervix. This is now removed from the algorithm' then that would go away. So, some of it's, how do we not have to be computer programmers to make that happen? But you have to be a computer programmer just to survive in healthcare these days, but that's just me."*
	Metrics penalize residency clinics	*"And I think that's worse in the residency clinic because, by definition, we're transitioning out a third of our resident doctors every year, and typically our faculty are less available to the clinic. And so, you don't have ease for the patient to have that continuity, right? So that becomes, I think, more difficult in a residency clinic."*
	Metrics cause doctors to dismiss patients not at goal	*"We don't have this private practice where you can cherry pick your patients and send out everybody. You know, your blood pressure's out of control, you're going in 30 days. Here's your letter. A1C is too high. I'm sorry, I tried. You're gone.*<br>*(Another participant responded) "I saw that happen in 2015 in Washington when they started dismissing patients whose A1Cs were over seven. Period. End of story. You are not part of our practice because we have a value-based model that requires that our A1C levels are under 7."*<br>*(First participant) "That was an internal medicine practice in a small town in Tennessee where I worked, so we got them all rolled over into our FQHC, which was fine. We love taking care of patients, and we weren't so stuck on those numbers."*
**More examples that existing accountability metrics cause moral distress to physicians**		
Metrics cause moral distress to physicians	Metrics create ridiculous	*"I guess and if they're going to be judging us in certain ways, there's some that are*

© 2026 Young R et al. *JAMA Network Open*.

	judgments of physician work	*just outright ridiculous like, there's one, on one of our measures that we get penalized for patients who we've never even seen before, but because we're listed, I guess as their primary care doctor and they haven't had things done, it's reflecting poorly on us. [W]e've had the conversation before, like, you know, it's hard to be a good doctor without ever actually even seeing somebody [laughter] because and so to get … reprimanded from … this stuff affects us from a financial standpoint, it kind of blows your mind and it's come to that."*
	Patient satisfaction priorities penalize physicians for providing high-quality care	*A: "So because the patient was expecting a Rocephin injection and I didn't give it to them, I completed, you know my encounter with the patient, my visit. And when the patient left the office the patient called the office manager, complained right? They came to receive a Rocephin shot and then, you know, it was not given to them. So the office manager comes to me and lets me know this is what the patient paid for, and can I give the patient the Rocephin shot."*<br>*Q: So by implication, it doesn't sound like you felt comfortable telling the office manager no. That that there was some expectation that you that your practice or whatever the office manager told you.*<br>*A: "Yeah, so, I did say "no." I don't think that I said it just like that simple, right? Like, "no." I can't remember what I said, but um I was actually fired from that job."*
	Metrics cause physicians to ask patients to take actions the physicians know are unlikely to happen	*"It leads to trying to game the system a little bit … they have such high expectations. You have to try to find a way to meet them. … How like the prescription medication and the percent that we have to hit well. OK, just keep sending it and asking them. You may not take this, but can you pick it up?"*
**CREATING A BETTER ACCOUNTABILITY SYSTEM FOR PRIMARY CARE**		

© 2026 Young R et al. *JAMA Network Open*.

It is difficult to convert family physician work to measurable outcomes	There are many subtleties to patient care	*"They want objectivity so they can put all of us in a box and give us a rating and then use that information to justify how reimbursement comes. But medicine's not like that. Treating patients isn't like that. There are so many subtleties in every case, every patient. There's so much gray area. What do you do? What should you do? What's the best decision? … [A]ll those subtleties, all that subjective stuff, you can't put it in a box. You can't just check it off and say 'I did a good job.' Because I was able to check that box and that satisfied what this administrator wanted it to do. The nature of medicine to me isn't that, you just can't do it that way. So, I don't have the answer."*
	Many participants talked about positive aspects of primary care but could not convert that to a measurable outcome.	*"So I say I would say we are good at identifying problems early on. And sometimes, even though the solution will not be quick. … I think that also we have – because of our unique role – we have a better impact in our patients. We can impact the patients better than if they go to see specialists. Um and we know our patients, right? We know their, not only … medical conditions, we a lot of the times know the social conditions, the financial conditions. So we have this holistic view of the patient that we carry with us."*
**More thoughts on Solutions**		
	Medicare should apologize for allowing barriers to high-quality care.	*"One thing that could be done to correct this system is for Medicare to just stop for a second and say 'oops, we screwed up.' When we had the opportunity to get every provider in the nation on the same EHR, we fumbled the ball. And we've created all these disparate systems that don't communicate with one another."*
	Decrease administrative costs by decreasing oversight	*"How much money is spent on people who are tracking all these measures? How many administrative people behind the scenes in the offices, who have nothing to do with patient care, who have nothing to do with actually seeing that patients are, are*

		getting what they need and doing whatever tests and treatments they need? But they are definitely monitoring every button you click. Every, every decision you make, there's somebody then, judging that decision and that button that you clicked. All those people make a lot of money, all those people have benefits and the cost that goes into this administrative burden, uh, get rid of that."
	Adjust physician ratings based on patient panel characteristics	*"And there has to be some sort of like, adjustment for the population of people you're seeing, you know. If I limited my practice to non-diabetics less than the age of 50, I would be like, the best doctor in the world, right?"*
	Many participants did not answer the question of a better accountability system, but talked about getting more resources for the existing system	*"What we need are more, it's like therapists and psychologists and psychiatrists, people to help us with the mental aspects. That stuff is uh, it's it feels impossible to get."*
	Recognize patient continuity with the physician as a proxy for patient satisfaction	*"I when I feel like, 'OK, I've done a good job' it's like the kind of patient I'm thinking about may be a guy who's seen nine different doctors before and he's got all these uncontrolled conditions and I'm able to, like, sit with him and communicate and make sure he's coming back frequently enough and he feels comfortable with me based on the way I'm able to interact with him. Those are the kinds of things I think of that that you know, maybe one of my strengths, …"*
	Reward physician efforts, even if a patient chooses to not follow through with the recommendation	*"There's no way that they find a way to at least give the doctor credit for having at least broached the subject. And say we had a discussion. The patient has chosen not to do the study and so forth and so on. And if we document it the right way or find a way where we could document it and get credit for that, I think that should count just as*

© 2026 Young R et al. *JAMA Network Open*.

		much as the patient having gotten the study."

© 2026 Young R et al. *JAMA Network Open*.
