# Supplementary material for: Family Physicians’ Views of Who They Are Accountable To and Current Quality Metrics
Source: JAMA Netw Open. 2026 Apr 16;9(4):e269281. doi: 10.1001/jamanetworkopen.2026.9281 (PMC13087814; doi:10.1001/jamanetworkopen.2026.9281)
Supplement: Supplement 2. — Nonauthor Collaborators [file jamanetwopen-e269281-s002.pdf]

Supplemental Online Content: Nonauthor Collaborators
*First name, last name, and suffix (if applicable) are required and will appear in PubMed.

***Group Name(s): Residency Research Network of Texas (RRNeT) Investigators group**										
***First Name and Middle Initial(s)**	***Last Name**	***Suffix (eg, Jr, III)**	Academic Degrees	Institution	Location (city, state/province, country)	Role or Contribution, eg, chair, principal investigator	Group (if more than 1 Group listed in the byline) and/or Subgroup (eg, Steering Committee)			
Inez	Cruz		PhD	Dept. Family and Community Medicine, UT Health-San Antonio	San Antonio, TX	Director of RRNeT, helped write the initial protocol, was the principal contact with the IRB, conducted some of the interviews				
David S	Edwards		MD	Department of Family Medicine, Sports Medicine Fellowship, Texas Tech University Health Sciences Center	Lubbock, TX	Helped write the initial protocol				
Arindam	Sarkar		MD, FAAFP	Dept. Family Medicine, Baylor Colle	Houston, TX	Co-led a focus group for Houston academic faculty and recruited participants for an employed, non-academic group.				
Meredith L	Williamson		Phd, ABPP	Department of Primary Care and Rura	College Station, TX	Helped review the initial protocol and interview guide				
F David	Schneider		MD	Department of Family and Community Medicine, UT Southwestern Medical Center	Dallas, TX	Helped write the initial protocol				
Deepu	George		PhD, LMFT	Primary Care & Community Medici	Edinburg, TX	Helped write the initial protocol				
Bailey A	Perez		PhD, MPH,	Dept. Family and Community Medicine, UT Health-San Antonio in the early period of the study, now with the Texas Department of State Health Services	San Antonio, TX, now Austin, TX	Research Coordinator, helped write initial protocol and worked with the IRB				
