# Supplementary material for: Family Physicians’ Views of Who They Are Accountable To and Current Quality Metrics
Source: JAMA Netw Open. 2026 Apr 16;9(4):e269281. doi: 10.1001/jamanetworkopen.2026.9281 (PMC13087814; doi:10.1001/jamanetworkopen.2026.9281)
Supplement: Supplement 3. — Data Sharing Statement [file jamanetwopen-e269281-s003.pdf]

Data Sharing Statement

Young. Family Physicians' Views of Who They Are Accountable To and Current Quality Metrics. *JAMA Netw Open*. Published April 16, 2026. doi:10.1001/jamanetworkopen.2026.9281

Data
Data available: No

Additional Information
Explanation for why data not available: We did not include this proviso in our consent process to participants.
